# Improving penalty shoot-out performance in skilled youth soccer players: An imagery modality-based approach

**DOI:** 10.1016/j.jsampl.2025.100125

**Published:** 2025-12-09

**Authors:** Vincent Joseph-Jacques, Roland Seux, Laurent Dominique, Vanessa Hatchi, Nicolas Robin

**Affiliations:** a Laboratory ACTES (URp5-4), UFR STAPS, Université des Antilles, Campus Fouillole, BP 592, Pointe à Pitre Cedex, 97159, France; b French Football Federation, Centre Elite des Régions Françaises d’Amérique, Les Abymes, France; c IRISSE Laboratory (EA 4070), UFR des Sciences de l'Homme et de l'Environnement, Département STAPS, Université de la Réunion, Le Tampon, France

**Keywords:** Football, Imagery, Performance, Penalty kick, Perspective

## Abstract

**Background:**

Since their introduction in professional football, penalty shoot-outs represent key moments in matches. Motor imagery (MI) can improve soccer performance, but different MI modalities can be used to mentally simulate motor actions. This study aimed to evaluate whether the MI modality used by the players would influence their penalty shoot-out performance.

**Methods:**

Twenty youth skilled football players from the Elite Center of the French Regions of America (CERFA) voluntarily participated in this experiment (M_age_ ​= ​16.4 years). After an assessment of their MI ability, the participants completed 4 counterbalanced experimental sessions spread over 4 weeks, each including the completion of 5 penalty shoot-outs. The sessions consisted of a control condition (count-down and actual execution) and 3 MI conditions during which the players had to mentally imagine themselves performing a penalty shoot-out according to one of the MI modalities: Internal Visual Imagery (IVI), External Visual Imagery (EVI), or Kinesthetic Imagery (KI), before kicking. Number of goals scored, shooting accuracy and ball speed were measured and served as dependent variables.

**Results:**

The soccer players benefited from MI. Indeed, in both the IVI and KI conditions, their performances were significantly higher than in the no-MI (i.e., control) condition with respect to the total number of goals scored and shooting accuracy variables.

**Conclusions:**

Practically, we suggest skilled football players imagine, from an internal perspective, taking a successful penalty kick before shooting.

In football, penalty shoot-outs occur when the two teams are unable to break the tie after regulation time and extra time [1]. They take place in elimination-type tournaments, where the winner advances to the next round and the loser is eliminated [2]. A penalty shoot-out consists of five shots for each team, with each team taking turns.

The outcome of the penalty shoot-out therefore becomes synonymous with qualification or potential elimination of the entire team [3]. This set-piece phase focuses all the pressure on the participating players, from their personal expectations, their teammates and coaches, the public, and the stakes associated with the outcome of this shootout [4]. These circumstances therefore impose different constraints on the players who participate in it, particularly psychological pressure [5]. Different training methods can be used to improve penalty kick performance, such as repetition, use of feedbacks or mental strategies [6].

Mental strategies can be used to help players perform in these highly stressful situations [7], such as motor imagery (MI), which allows players to manage their emotions and optimize their performance [8,9]. MI can be defined as the ability of a human brain to resynthesize motor experiences without any overt movement [10]. According to Decety and Jeannerod [11], MI is a conscious and dynamic state during which the representation of a specific motor action is reactivated in the brain in the absence of actual movement. MI and actual performance are functionally similar [12]. By mobilizing the neural networks responsible for the corresponding motor action, MI can strengthen these circuits and facilitate motor learning and the improvement of technical gestures [9]. Thus, this mental strategy is frequently used in the sports field [13,14], among both amateur and professional athletes, in order to improve motor performance [15]. According to Toussaint and Blandin [16], there are three main types of MI modalities used in the context of sports practice: Internal visual imagery (IVI; seeing the action through one's own eyes), external visual imagery (EVI; seeing the action filmed by a camera) and kinesthetic imagery (KI; feeling the sensations related to the performance of motor actions).

Research has shown the beneficial effects of MI used alone compared to a control condition [17], however, the improvement in motor performance was generally lower than that obtained after actual practice [15]. In addition, many studies have shown greater positive effects when MI was combined with actual execution of motor actions [18,19]. However, Toussaint and Blandin [16] demonstrated that the improvement of motor performance can depend on the MI modality used. Indeed, White and Hardy [20] reported modality-specific effects of visual MI in the context of a motor task constrained by environmental variability. The authors investigated the influence of IVI and EVI on the acquisition of a motor skill that relied on environmental signal processing (i.e., a slalom task). The results indicated that participants who employed IVI committed fewer spatial errors during a transfer task (i.e., a novel course), compared to those who used EVI. On the contrary, EVI has demonstrated beneficial effects in motor tasks that emphasize the accurate reproduction of body postures such as karate katas [9,21]. In a study realized by the latter authors, 25 expert karate athletes were instructed to learn a new kata using either IVI or EVI modalities, and findings revealed a significant advantage for the EVI group. Comparable results were also found among novice athletes learning a gymnastics sequence [21] or improving their pass skill performance in football tasks [22,23]. Finally, KI appears to facilitate motor tasks requiring intersegmental coordination [18] and muscular strength [24] such as serving or kicking. As mentioned by Ridderinkhof and Brass [25], KI engages internal anticipatory representations of action outcomes and is associated with predictive motor control. This internal simulation mechanism is proposed to enhance motor performance through emulative processes [26].

Given the differentiated effects of MI modalities on performance in the different motor tasks previously mentioned, it seems important to determine if one or more imagery modalities would be the most appropriate, in a football precision shooting task, to obtain the highest performance. In the field of football, while some research has not shown significant performance improvement following MI intervention (e.g., [27,28]) others have observed positive effects (e.g., [7,23,29,30]) but without specifying the type of MI modalities used in the mental simulation of the penalty shoot-out, or without distinguishing IVI from EVI whereas differentiated effects were observed in other motor precision tasks (e.g., [18]).

This study aimed to identify the potentially differentiated effects of MI modalities (i.e., internal visual, external visual, and kinesthetic imageries) on penalty shoot-out performance in skilled youth soccer players. Consistent with previous research, the primary objective of the current study was to assess whether the players should benefit from MI; and the secondary objective was to determine whether this improvement might also be a function of the imagery modality used during the pre-shooting MI.

## 1 Methods

### 1.1 Participants

An a priori power analysis (G∗Power 3.1) was used (effect size 0.35; alpha 0.05 and power 0.95) to calculate the total sample size (*n* ​= ​19, with critical F ​= ​2.77 and actual power 0.95).

Twenty skilled soccer players, aged between 15 and 17 (M_age_ ​= ​16.4 years, SD ​= ​0.86), playing at the Elite Center of the French Regions of America (CERFA) volunteered to participate in this study. They had been playing soccer for over 10 years, trained 5 days a week and competed in regional and national competitions. All participants, as well as their legal representatives, signed a consent form. This study, validated by the ethics committee of our laboratory (ACTES URp5-4-2024-11), was conducted in accordance with the latest version of the Declaration of Helsinki (1964).

### 1.2 Material and tasks

This study took place at the CERFA training grounds. The sessions were conducted on an approved synthetic pitch. The goal cages were standard size (7.32 ​m ​× ​2.44 ​m). The footballs, Nike Aerowsculpt Flight, were inflated between 600 and 700 ​g/cm3 in accordance with competition standards. During the sessions, the players used their same outfit and personal individual equipment (cleats, shin guards and gloves for goalkeepers).

All players completed the Movement Imagery Questionnaire third French version (MIQ-3f; [31]). This questionnaire consists of 12 items that assess the ease or difficulty of performing IVI, EVI, and KI. Each item of the MIQ-3f corresponds to a single leg, arm, or whole-body movement performed physically before being mentally simulated in the specified MI modality. The soccer players then rated the difficulty or ease they had forming the mental representation of the movement using two 7-point Likert-type scales (ranging from 1 [very difficult to feel/see] to 7 [very easy to feel/see]), referring, respectively, to the KI and IVI or EVI, modalities.

The penalty shout-out test consisted of performing five penalty kicks for each of the participants [32]. The number of goals scored was recorded. All penalty shots were filmed with a camera (Panasonic HC-V777) with a tripod placed behind the shooter [33] for image capture and shot analysis, and the speed of the balls was measured with a radar gun (SR 3600).

### 1.3 Procedure

Before the start of the experimental phase, participants and their legal guardians completed consent forms for participation in the experiment. Then, all players fulfilled the MIQ3f [6] to assess their MI ability (see [18] for a similar procedure).

During the experimental session spread over 4 weeks, the participants had to complete a penalty shoot-out session integrated into their regular training sessions. For organizational reasons, participants were randomly assigned to 4 groups to perform the 4 counter-balanced sessions (Control vs. IVI vs. EVI vs. KI; see Table 1).

**Table 1:** Time course of the experimental design.

Pre experimental			Experimental counterbalanced sessions			
Questionnaire	Imagery ability	Subgroups	Week 1	Week 2	Week 3	Week 4
Inform consent	MIQ-3f	1	IVI	EVI	KI	Control
Inform consent	MIQ-3f	2	EVI	KI	Control	IVI
Inform consent	MIQ-3f	3	KI	Control	IVI	EVI
Inform consent	MIQ-3f	4	Control	IVI	EVI	KI

IVI (internal visual imagery), EVI (external visual imagery), KI (kinesthetic imagery).

Each experimental session began with a standardized collective warm-up lasting 15 ​min. Participants then took turns taking 5 shots on goal. The shooters took turn shooting at one of the 4 goalkeepers. These alternated every five attempts and were counterbalanced between the players (for a similar procedure see Joseph-Jacques et al., revised). For each of the 5 shots, players had to follow the instructions specific to each of the experimental conditions.

During the Control condition, participants were instructed to take the penalty kicks after completing a mental counting-down task. After placing the ball and then stepping back to take their run-up steps, each player had to count down in English from 5 to 0, just before kicking the ball. At the end of the session, the experimenter asked them if they had used a mental technique before shooting (see [34] for a similar procedure).

During the KI condition and before each actual shot, the players had to imagine feeling the sensations of strength, muscle contraction and relaxation, speed or contact with the ball that they generally feel before shooting a penalty.

During the EVI condition, and before kicking, the players had to imagine themselves as if they were seeing themselves in the third person (or as if they were filmed with a camera placed on their side).

Finally, during the IVI condition, and before each real execution, the participants had to imagine, as if they were seeing themselves (first person perspective) through their own eyes (or as if they had a “Go Pro” on their head) performing their penalty shout-out (see [18] for a similar procedure).

At the end of each session including MI, players were asked to rate the quality of the mental images produced using the Imagery Quality Index, comprising a Likert scale ranging from 1 (“*Unclear and weak mental representation*”) to 7 (“*Perfectly clear and vivid mental representation*”); (for a similar procedure see [18]).

### 1.4 Data analysis

One participant's data was not considered because he was injured, leaving a final sample of 19 players.

Participants’ imagery ability was measured to ensure that the sample did not contain participants having difficulties performing MI [35]. The psychometric properties of the MIQ-3f (internal consistency: reliability score ≥0.88 for the three subscales) and test-retest reliability (intraclass correlation coefficients: 0.87 for IVI, 0.86 for IVE, and 0.88 for IK) were found to be satisfactory [6].

The number of goals scored and the shooting accuracy score served as dependent variable. The later dependent variable, established by Rhodes et al. [36], corresponded to: 2 points for a goal scored, 1 point for a shot on target stopped by the goalkeeper and 0 points for a shot off target. An additional shooting accuracy score, corresponding to 3 points, was awarded for goals scored in one of the “unstoppable” zones (see [36] for a similar procedure). These zones corresponded to the two top corners and the areas located at the foot of the goalposts, which measured 70 ​cm on each side [7,37]. These zones were specified to the participants before the start of the experiment (for a similar procedure see [38,39]).

Participants' scores on the MIQ-3f questionnaire [6], vividness scores of mental images produced during the MI, and shot speeds in km/h (recorded during the different test conditions and carried out using the sports radar gun) served as check variables (see [40] for a similar procedure).

Before performing the analyses of variance (ANOVAs), the normality of distribution (Kolmogorov–Smirnov test) and the homogeneity of variances (Levene test) were checked. Experimental conditions (Control vs. IVI vs. EVI vs. KI) repeated-measures ANOVAs were calculated for each of the dependent variables (i.e., number of goals scored and shooting accuracy score) and for the shot speed variable. An alpha threshold was set at 0.05 for all analyses, effect sizes (ηp2) were reported, and post hoc analyses were performed using Bonferroni tests.

## 2 Results

### 2.1 Imagery ability

None of the participants reported difficulty performing MI. The players reported having vivid and clear images before shooting (M ​= ​5.2). They could be considered “good imagers” [35] according to their MIQ-3f scores (M_IVI_ ​= ​5.18, M_IVE_ ​= ​5.23, M_KI_ ​= ​5.18). No players reported using MI in their personal practice in training or matches.

### 2.2 Number of goals scored

**Fig. 1:**
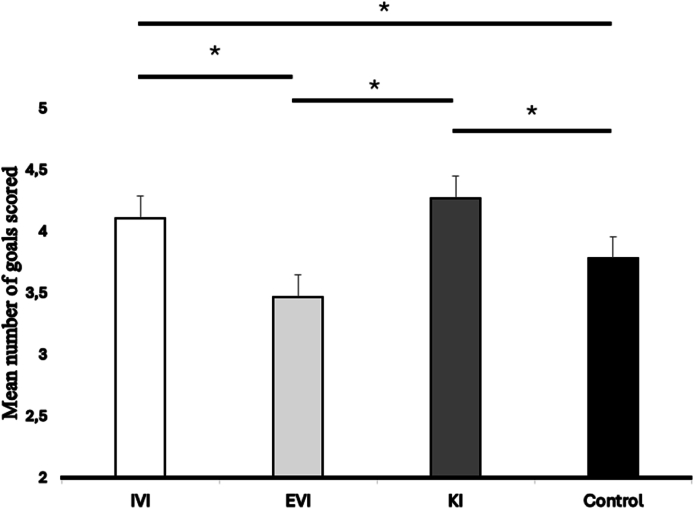
Mean number of goals scored according to each experimental condition (∗*p* ​< ​0.05). IVI (internal visual imagery), EVI (external visual imagery), KI (kinesthetic imagery).

The ANOVA computed on the number of goals scored revealed a significant main effect of condition [F(3, 54) ​= ​3.29, *p* ​< ​0.05, η_p_^2^ ​= ​0.15]. As illustrated in Fig. 1 and Table 2, the post hoc test revealed that the players scored more goals in the IVI (M ​= ​4.11) and KI (M ​= ​4.27) conditions compared to the IVE (M ​= ​3.47) and Control conditions (M ​= ​3.78).

**Table 2:** Mean number of goals scored and shooting accuracy scores according to each experimental condition: IVI (internal visual imagery), EVI (external visual imagery), KI (kinesthetic imagery) and Control. (∗*p* ​< ​0.05).

Conditions	IVI	IVE	KI	Control
Mean number of goals scored (*SD*)	4.11∗ (*0.23*)	3.47 (*0.15*)	4.27∗ (*0.28*)	3.78 (*0.16)*
Mean shooting accuracy scores (*SD*)	1.98∗ (*0.12*)	1.69 (*0.09*)	1.92∗ (*0.10*)	1.78 (*0.13*)

### 2.3 Shooting accuracy scores

**Fig. 2:**
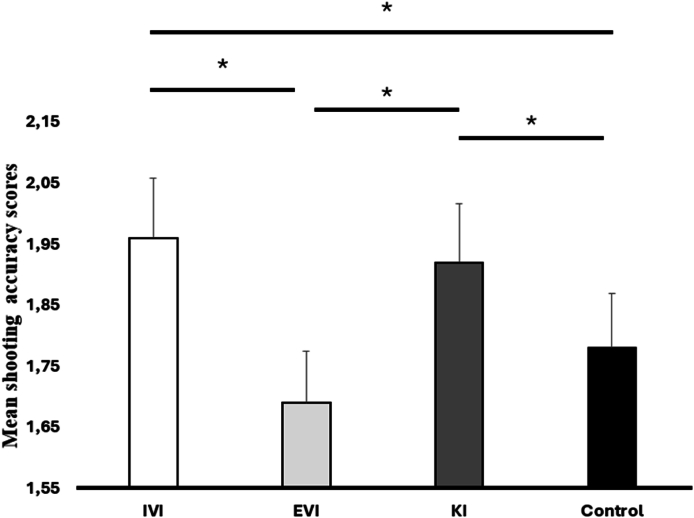
Shooting accuracy scores according to each experimental condition (∗*p* ​< ​0.05). IVI (internal visual imagery), EVI (external visual imagery), KI (kinesthetic imagery).

The ANOVA computed on the shooting accuracy scores revealed a significant main effect of condition [F(3, 54) ​= ​3.47, *p* ​< ​0.05, η_p_^2^ ​= ​0.17]. As illustrated in Fig. 2 and Table 2, the post hoc test revealed that the players had higher mean accuracy scores in the IVI (M ​= ​1.98) and KI (M ​= ​1.92) conditions compared to the IVE (M ​= ​1.69) and Control conditions (M ​= ​1.78).

### 2.4 Shot speed

The analysis revealed no main effect of condition [F(3, 54) ​= ​1.31, *p* ​= ​.28, η_p_^2^ ​= ​0.06], which means no statistical differences between the penalty shoot-out speeds measured during the 4 experimental sessions.

## 3 Discussion

The aim of this study was to evaluate, on one hand, whether MI could improve penalty-shootout performance in soccer players and, on the other hand, whether this improvement was modulated by the MI modalities used in mental simulations of shots performed before the actual execution.

In the context of MI, visual and kinesthetic imagery are the most frequently used modalities due to their decisive role in optimizing motor performance [41]. As hypothesized, the soccer players benefited from MI. Indeed, the scores obtained by the participants, particularly during the IVI and KI conditions, were significantly higher than in the no-MI (i.e., Control) condition regarding the total number of goals scored and the shooting accuracy variables. These results are consistent with previous research on the use of MI to enhance athletic performance (e.g., [[42], [43], [44]]). Studies have shown that participants who received MI intervention improved their sports performance (for reviews, see [14,15]), particularly in football (e.g., [7,22,23]), compared to a control group that did not perform MI. However, and unlike studies that did not observe performance improvements following an MI intervention (e.g., [27,28]) or that did not specify the type of MI modality to be used during imagined actions [7,23,29,30], the results of the present study highlight that the MI modalities that seem best suited to a penalty-shootout task are IVI and KI.

This result is consistent with the results found by Dominique et al. [18] who observed that skilled tennis players had higher service performance when using IVI and KI modalities. As evoked by Guillot [9], the use of KI may be particularly beneficial when participants have some degree of expertise in a task as it is easier to imagine sensations of force, relaxation or contraction of controlled movement. In addition, topokinetic activities (e.g., goal- or target-oriented, such as throwing, serving in racket sports, or set pieces in soccer) appear to benefit from IVI [45].

Furthermore, authors argued that IVI and KI are based on the activation of anticipatory images of the effect of the movement [25] and are linked to predictive motor control [18]. During IVI and KI, the premotor cortex, which involves the planning and preparation of actual motor action, is activated [[46], [47], [48]] and the internal simulation of the movement may enhance performance. Moreover, these two modalities are intrinsically bound, especially due to concomitant motor and visual experience which is gained when performing the actual task. Due to this, it could be that both internal modalities are experienced at the same time.

However, it is also important to highlight that the results of the current study showed that EVI was the least effective of the three MI modalities with respect to performance in the penalty-shootout task. This result can be explained by the fact that external visual imagery is best suited for learning and realizing tasks that rely heavily on form for their successful performance [20]. This result is in line with Dominique et al. [18] study, in which the EVI had not improved service performance. It seems that EVI is more beneficial in novices and non-expert soccer players [22]. Indeed, Hardy and Callow [21] proposed that an external visual perspective may benefit learning tasks which are more gross body movements and where form might be most important. Moreover, once they have developed a certain degree of expertise in the task, as is the case of the football players who participated in the current study, it seems that players prefer to use internal MI perspectives, which are centered on themselves [18], rather than external perspectives (i.e., EVI), which consist of imagining the movement as a spectator [49,50]. Moreover, some research suggests that prior visual experience contributes to the ability to imagine in the visual modality [51]. It is possible that the participants in the current study had more experience with the first-person perspective (internal visual) than with watching themselves on video performing taking penalties (corresponding to the external visual or third perspective). In addition, it could be that instructing this third person perspective in experienced individuals results in a declarative breakdown of the action sequence and induces reinvestment-like processes [52]. Finally, it is also important to note that when individual preference differs from that induced by the demands of the discipline, it is often the former that is generally favored [45]. It would therefore be interesting, in future research, to collect the players' IM preferences [18] and then assess whether these would allow optimal performance to be obtained in order to allow individualization of instructions.

This study is not without limitations. The first one concerns the training context in which the experiment was conducted, which limited the pressure felt by the players and the stakes related to their shooting performance. It is important to highlight that the current study was performed, in ecological condition, during regular CERFA's training sessions and therefore the participants' anxiety and stress levels were lower than in a real competitive match. The second concerns the training age of the players and their training status, which can vary from one participant to another. Additionally, although the MIQ-3 scores appear to indicate that none of the participants had difficulty performing MI [35], players reported that they did not use MI in their personal practice, and it is possible that this mental task interfered with their shot preparation. However, players improved their penalty shoot-out performance by using KI and IVI before shooting and performed better than in the control condition. It would therefore be relevant to implement an MI intervention with players to accustom them to the penalty shootout simulation task in order to confirm the results of this study. Finally, since switching between different forms of IM is common in visualization instructions, it is essential to develop them, not necessarily identically, but sufficiently to be able to use them alternately [45].

As a research perspective, in future studies, it would be interesting to measure the effect of players' imagery modality preferences and to integrate these into their pre-performance shooting routine which would consist of a standardized number of swing steps, then deep breathing and the MI of a successful shot as recommended in tennis (see [43]). Further research will be conducted in our laboratory to test these hypotheses.

## 4 Conclusion

Consistent with previous studies showing the beneficial effect of using MI before performing a motor action, the main results of this original study revealed that the soccer players had higher penalty-shootout performance when using KI and IVI compared to the Control condition but also compared to EVI. We recommend skilled football players to have recourse to the mental simulation, rather from internal perspectives, of a successful shot before kicking the ball in a penalty-shootout in match conditions. We also recommended that football coaches use MI, integrated into training, to improve penalty-shootout performance in young players.

## Founding declaration

This research did not receive any specific grant from funding agencies in the public, commercial, or not-for-profit sectors.

## Declaration of competing interest

The authors declare that they have no known competing financial interests or personal relationships that could have appeared to influence the work reported in this paper.
